# Transcranial Direct Current Stimulation Modulates Neurogenesis and Microglia Activation in the Mouse Brain

**DOI:** 10.1155/2016/2715196

**Published:** 2016-06-15

**Authors:** Anton Pikhovych, Nina Paloma Stolberg, Lea Jessica Flitsch, Helene Luise Walter, Rudolf Graf, Gereon Rudolf Fink, Michael Schroeter, Maria Adele Rueger

**Affiliations:** ^1^Department of Neurology, University Hospital of Cologne, Kerpener Strasse 62, 50924 Cologne, Germany; ^2^Max Planck Institute for Metabolism Research, Gleueler Strasse 50, 50931 Cologne, Germany; ^3^Cognitive Neuroscience, Institute of Neuroscience and Medicine (INM-3), Research Centre Juelich, 52425 Juelich, Germany

## Abstract

Transcranial direct current stimulation (tDCS) has been suggested as an adjuvant tool to promote recovery of function after stroke, but the mechanisms of its action to date remain poorly understood. Moreover, studies aimed at unraveling those mechanisms have essentially been limited to the rat, where tDCS activates resident microglia as well as endogenous neural stem cells. Here we studied the effects of tDCS on microglia activation and neurogenesis in the mouse brain. Male wild-type mice were subjected to multisession tDCS of either anodal or cathodal polarity; sham-stimulated mice served as control. Activated microglia in the cerebral cortex and neuroblasts generated in the subventricular zone as the major neural stem cell niche were assessed immunohistochemically. Multisession tDCS at a sublesional charge density led to a polarity-dependent downregulation of the constitutive expression of Iba1 by microglia in the mouse cortex. In contrast, both anodal and, to an even greater extent, cathodal tDCS induced neurogenesis from the subventricular zone. Data suggest that tDCS elicits its action through multifacetted mechanisms, including immunomodulation and neurogenesis, and thus support the idea of using tDCS to induce regeneration and to promote recovery of function. Furthermore, data suggest that the effects of tDCS may be animal- and polarity-specific.

## 1. Introduction

Transcranial direct current stimulation (tDCS) can be used to induce alterations of cortical excitability in a polarity-specific way, in both animals and humans [1, 2]. Effects on cortical excitability outlast the actual stimulation (“after-effects”) and include NMDA-receptor dependent synaptic plasticity [3]. Clinical data suggest that tDCS may facilitate rehabilitation after cerebral ischemia [4–6]. However, data are inconsistent and the neurobiological mechanisms underlying tDCS remain poorly understood, impeding its implementation into clinical routine [7].

Cerebral ischemia induces various processes at the cellular level, including the activation of brain-resident microglia (“neuroinflammation”) as well as the mobilization of neural stem cells from their niches [8–11]. In the rat, tDCS applied with a sublesional current intensity activates innate immune responses and mobilizes neural stem cells, suggesting that the application of tDCS after cerebral ischemia may promote recovery of function by facilitating regeneration [12, 13]. With the perspective to investigate tDCS effects in genetically modified mice, we recently established tDCS in the mouse [14]. Under the hypothesis that, analogous to the rat, both immune and stem cells are affected by tDCS in the mouse, here we investigated the effects of multisession tDCS on resident microglia as well as on endogenous neural stem cells in the mouse brain.

## 2. Material and Methods

### 2.1. Animals and Surgery

**Figure 1:**
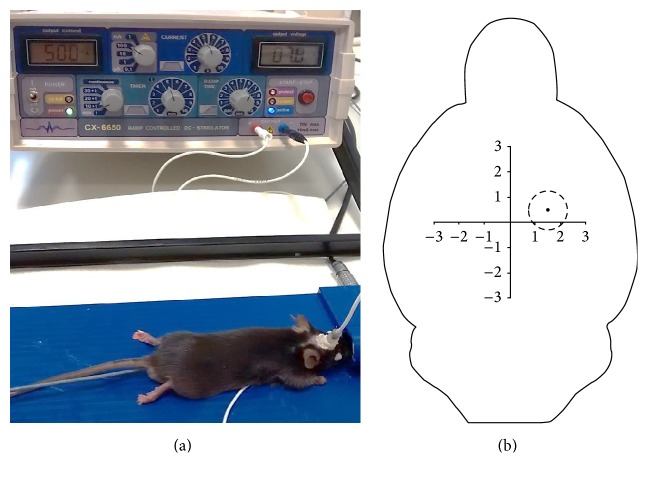
Experimental setup for tDCS. (a) The anesthetized mouse was connected to the direct current stimulator (apparatus in the back) via a silver-coated electrode cable attached to the polycarbonate tube mounted on its skull. The cable protruding from under the mouse' abdomen originates from the counter electrode. (b) The epicranial electrode (dashed circular line) was mounted on the intact skull using dental cement at the coordinates AP +0.5 mm and ML +1.5 mm from bregma.

All animal procedures were approved by the local animal care and use committee and governmental authorities (LANUV, # 84-02.04.2013.A068). Surgery was performed on twenty 10–12-week-old male C57BL/6JRj mice (Janvier Labs, France), weighing 28–35 g, under light isoflurane anesthesia, and additional local anesthesia with bupivacaine. To ensure identical electrode placement for tDCS, custom-made polycarbonate tubes with a contact area of 2.27 mm^2^ (Medres Medical Research, Cologne, Germany) were stereotactically placed on the skull of the mice prior to tDCS, as described previously [14]. In brief, mice were placed in a stereotactic frame, a small incision was made in the skin of the head, and the skull was dried with cotton swabs. A polycarbonate tube was stereotactically placed on the intact skull at bregma AP +0.5 mm, ML +1.5 mm, and subsequently attached to the bone surface with a thin layer of nontoxic dental cement (Super-Bond C&B, Sun Medical, Japan) and a second layer of two-component luting resin (Ketac Cem Plus, 3MESPE AG, Germany). To ensure current flow during stimulation, the hollow implant was kept free of cement. To avoid debris accumulating in the polycarbonate tube, a custom-made screw cap sealed the device when not used.  Figure 1 provides an overview of the experimental setup. After surgery, mice were transferred back to their home cage and had access to food and water ad libitum.

### 2.2. TDCS

Animals were randomized to receive 10 days of tDCS with either cathodal or anodal polarity; a third group of mice was not stimulated for control (sham group). Additionally, mice were randomized to receive different currents of tDCS, 250 *µ*A or 500 *µ*A, resulting in 5 different experimental groups of *n* = 4 mice each. Charge (*Q*) and contact area (*A*) were used to calculate the applied charge density *σ* as *σ* = *Q*/*A*, resulting in charge densities of either 99.118 C/m^2^ or 198.237 C/m^2^, respectively.  Table 1 provides an overview of the experimental groups.

TDCS was repeated daily for 5 consecutive days, followed by a tDCS-free interval of 2 days; then animals were subjected to tDCS for 5 more days, resulting in 10 days of tDCS in total. This experimental design was adapted from clinical studies with stroke patients. For each tDCS session, the polycarbonate tube was filled with saline, a silver-coated tDCS electrode (Medres Medical Research, Cologne, Germany) was inserted, and a silver-coated sensor electrode (Spes Medica, Italy; #DENIS01526) was placed under the shaved thorax as counter electrode. TDCS was applied continuously for 15 minutes using a constant current stimulator (CX-6650, Schneider-Electronics, Gleichen, Germany) under light isoflurane anesthesia. In the control group, mice were anesthetized for 15 minutes without connection to the stimulator (“sham”). After each tDCS session, animals were allowed to recover in their home cages with access to food and water ad libitum.

On every second day of tDCS or sham stimulation, respectively, animals received intraperitoneal injections of bromodeoxyuridine (BrdU; Sigma-Aldrich, Munich, Germany) at a concentration of 100 mg/kg, in order to label proliferating cells.

### 2.3. Immunohistochemistry

Mice were euthanized by decapitation two days after the last tDCS session. Brains were removed rapidly and frozen at −80°C until used. Frozen brains were cut in coronal sections of 10 *µ*m and fixed with 4% paraformaldehyde. To exclude any animals with tDCS-induced cortical lesions, sections were stained for neuronal integrity using the neuronal marker NeuN (mouse monoclonal, 1 : 2000, cat# MAB377, Millipore, Germany). In order to assess the effect of tDCS on local inflammation, activated resident microglia expressing the ionized calcium binding adaptor molecule 1 (Iba1) were labeled with polyclonal rabbit anti-Iba1 (1 : 1000, cat# 019-19741, Wako, Germany). To quantify effects of tDCS on neurogenesis, neuroblasts were stained against doublecortin (DCX; goat polyclonal, 1 : 800, cat# sc-8066, Santa Cruz Biotechnology, USA). Proliferating cells were stained against bromodeoxyuridine (BrdU; rat monoclonal, 1 : 300, cat# ab6326, Abcam, UK). For visualization, the ABC-Elite kit (Vector Laboratories, USA) with diaminobenzidine (Sigma, Germany) as final reaction product was used. Fluoro-Jade C staining was performed to reveal neuronal degeneration according to manufacturer's protocols. In brief, tissue was pretreated with 1% sodium hydroxide in 80% alcohol and then with 0.06% potassium permanganate. Next, 0.0004% Fluoro Jade C (cat# AG325-30MG, Millipore, Germany) in 0.1% acetic acid was applied.

Image analysis and data quantification was performed by an independent observer blinded to the treatment conditions. To quantify DCX-positive neuroblasts or BrdU-positive proliferating cells in the SVZ, the area covered by immunoreactive cells was measured in *μ*m^2^ using the software ImageJ (Version 1.84, NIH), analyzing 6 tissue slices at 100 *µ*m intervals. To quantify the number of NeuN-positive neurons and Iba1-positive microglia in the cortex, images from the respective stainings were taken throughout the brain at 100 *µ*m intervals. Using the ImageJ software, an area of interest was defined throughout the entire cortex at coordinates ML +3 to −3 mm related to Bregma. Immunoreactive cells were counted as cells per mm^2^ and mean values generated per mm^2^ for each mouse.

For all quantifications, mean values were established among equally treated mice.

### 2.4. Statistical Analyses

Descriptive statistics, calculating means and standard errors, were performed with Microsoft Excel 2010 (Microsoft Corp.). All other statistical analyses were performed with the software Prism (Version 6.01, GraphPad, USA). For comparison of multiple groups, multifactor Analysis of Variance (ANOVA) was performed, followed by Tukey's Honest Significant Difference (HSD) test. If data were not normally distributed, an ANOVA on ranks was performed, followed by Dunn's multiple comparisons test as post hoc analysis. Statistical significance was set at the less than 5% level (*p* < 0.05).

## 3. Results

### 3.1. Multisession tDCS at a High Charge Density Causes Cortical Lesions

**Figure 2:**
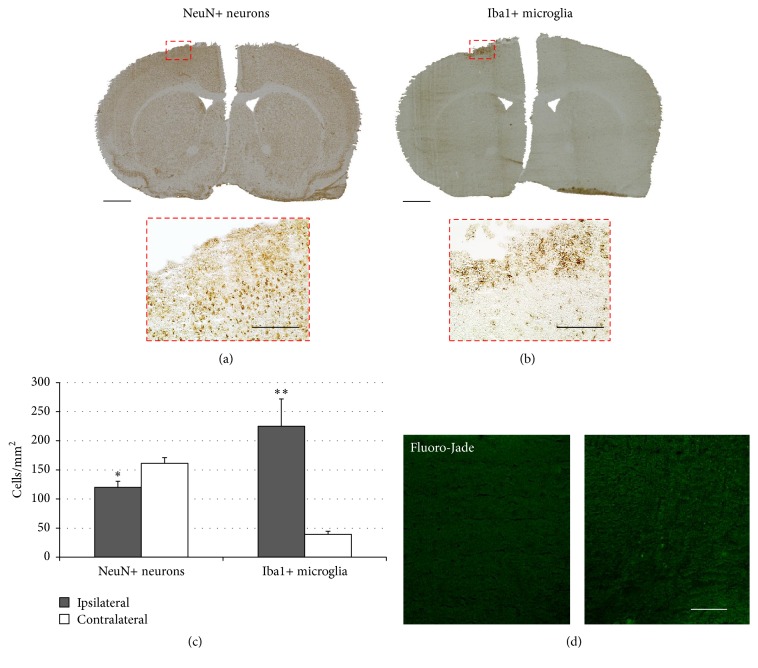
Multisession transcranial direct current stimulation (tDCS) at a high charge density causes cortical lesions. (a) After multisession tDCS with 198 kC/m^2^, several animals presented with a disruption of neuronal integrity on NeuN neuronal staining. The lower image depicts the magnified region from the upper image. The scale bars represent 1 mm (upper image) and 200 *µ*m (lower image), respectively. (b) In focal cortical lesions identified by NeuN-staining, an accumulation of Iba1-positive activated microglia indicated local inflammation. Again, the lower image depicts the magnified region from the upper image; scale bars represent 1 mm (upper image) and 200 *µ*m (lower image), respectively. (c) Quantification confirmed that in focal cortical lesions induced by tDCS of high charge density, loss of NeuN-positive neurons was accompanied by upregulation of Iba1-positive microglia (^*∗*^ *p* < 0.05, ^*∗∗*^ *p* < 0.01). (d) Left: Fluoro-Jade staining depicts intact cortical tissue following tDCS with 99 kC/m^2^. Right: small cortical lesion with Fluoro-Jade-positive degenerating neurons; scale bar represents 100 *µ*m.

TDCS was applied repetitively for 10 consecutive days at two different charge densities (Table 1). In mice stimulated with the higher charge density of 198 kC/m^2^, a disruption of neuronal integrity was observed in 3 out of 4 anodally and 1 out of 4 cathodally stimulated animals (50% of mice in total). These focal and superficial cortical lesions were located directly under the polycarbonate tube implanted onto the intact skull for tDCS (Figure 2(a)). Cortical lesions were accompanied by an upregulation of activated microglia in the very same area, suggesting an acute inflammatory reaction (Figure 2(b)). Quantification showed a significant decrease in viable neurons within the lesion of the affected animals (*p* < 0.05, *t*-test), along with an over fivefold increase in Iba1-positive activated microglia (*p* < 0.01, *t*-test; Figure 2(c)). Additionally, neurodegeneration was assessed by Fluoro-Jade staining, showing intact cortical tissue in mice stimulated with 99 kC/m^2^ (Figure 2(d)).

Since at the low charge density of 99 kC/m^2^, no impairment of neuronal integrity or any other sign of cortical lesions were observed in any of the experimental animals. Thus, further immunohistochemical analyses were exclusively conducted on mouse brains stimulated with 99 kC/m^2^.

### 3.2. Multisession Anodal tDCS at Low Charge Density Downregulates the Constitutive Expression of Iba1 by Microglia

**Figure 3:**
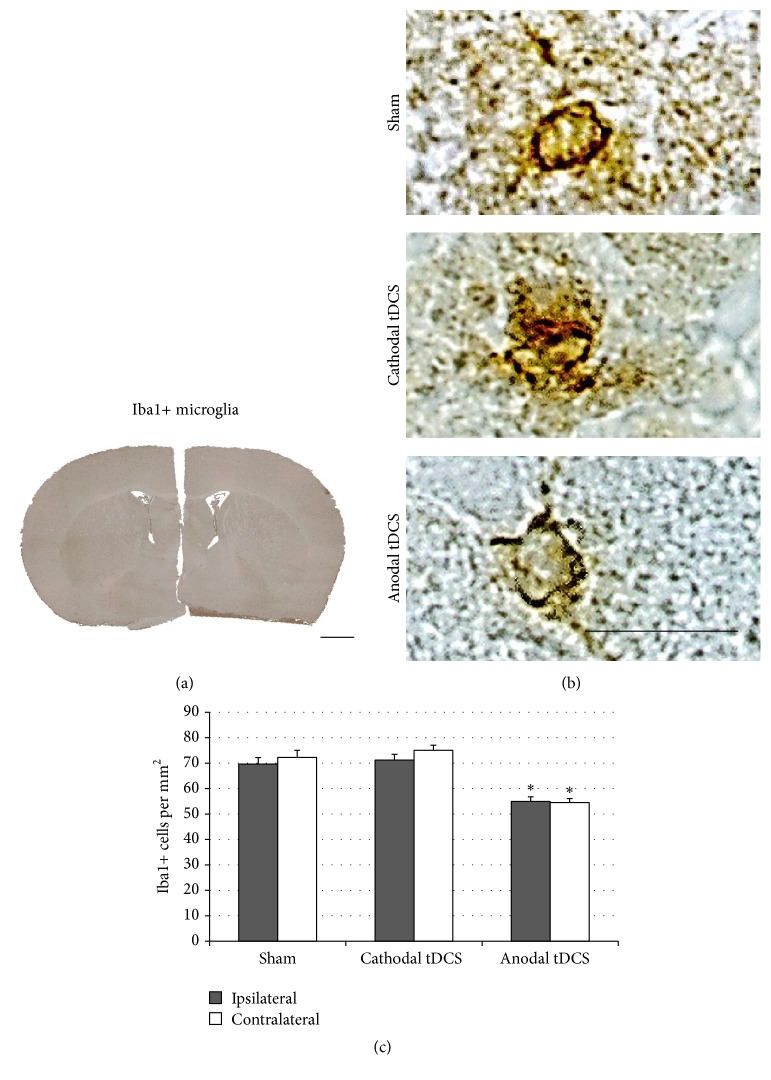
Multisession anodal tDCS at low charge density downregulates the constitutive activation of microglia. (a) Multisession tDCS with 99 kC/m^2^ did not cause focal cortical lesions (scale bar represents 1 mm). (b) Microglia expressing Iba1 displayed an amoeboid morphology irrespective of stimulation polarity (scale bar represents 20 *µ*m). (c) The number of Iba-positive microglia in the cortex was significantly decreased following multisession anodal tDCS both ipsilateral and contralateral to stimulation (^*∗*^ *p* < 0.05).

Mice stimulated at the low charge density of 99 kC/m^2^ were stained for Iba1 to assess and quantify activated microglia. Iba1+ microglia were found equally distributed throughout the cortex, without any focal areas of microglia accumulation (Figure 3(a)). Microglia expressing Iba1 displayed an amoeboid morphology irrespective of the presence or polarity of stimulation (Figure 3(b)). To assess the effect of tDCS on this constitutive expression, Iba1-positive cells were counted throughout the cortex of both the tDCS-stimulated (ipsilateral) hemisphere as well as the contralateral hemisphere. After 10 days of anodal tDCS, the number of Iba1+ microglia was significantly reduced in both the ipsilateral and the contralateral cortex, indicating that anodal tDCS downregulated the constitutive expression of Iba1 by cortical microglia (*p* < 0.05 [Dunn's]; Figure 3(c)).

### 3.3. TDCS Induces Neurogenesis in the SVZ

**Figure 4:**
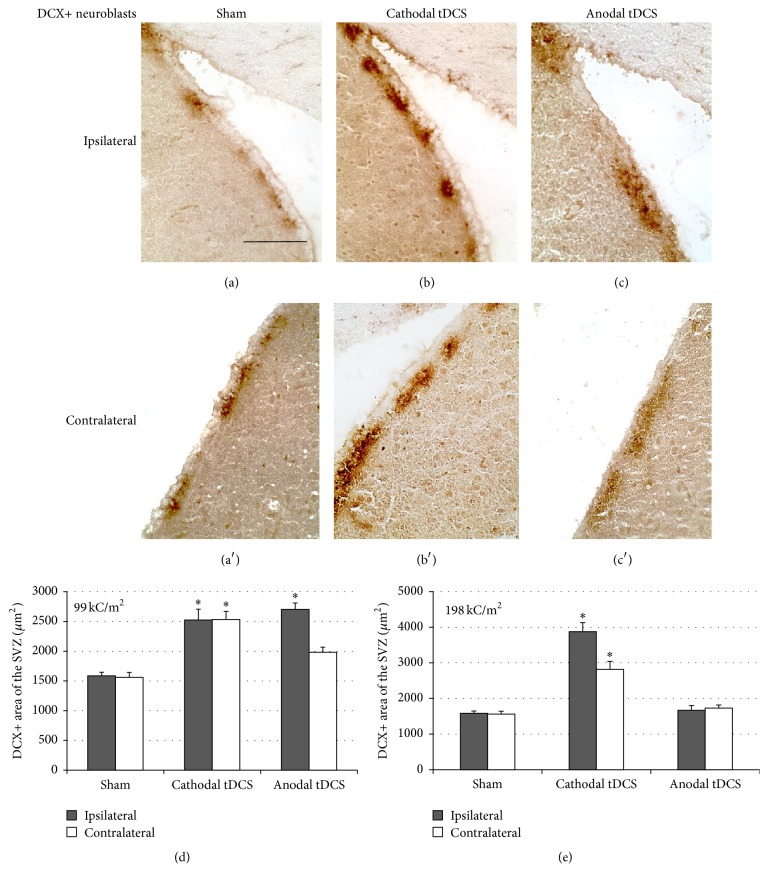
Multisession tDCS induces neurogenesis in the subventricular zone (SVZ). (a), (a′) Neuroblasts in the SVZ were identified by their expression of DCX under control conditions (sham). The scale bar represents 100 *µ*m and applies to panels (a)–(c′). (b), (b′) Multisession cathodal tDCS with 99 kC/m^2^ increased DCX-immunoreactivity in the SVZ. (c), (c′) Following multisession anodal tDCS with 99 kC/m^2^, the area of DCX-positive neuroblasts in the SVZ ipsilateral to tDCS was wider than under control conditions (sham). (d) Quantification confirmed that multisession cathodal tDCS with 99 kC/m^2^ increased the size of the DCX-immunoreactive area in the ipsilateral and contralateral SVZ, while multisession anodal tDCS with 99 kC/m^2^ increased the size of this area ipsilateral to stimulation only (^*∗*^ *p* < 0.05). (e) In animals stimulated with 198 kC/m^2^ that did not display any lesion, neuroblasts were increased in the ipsi- and contralateral SVZ after cathodal tDCS. Anodal tDCS with 198 kC/m^2^ did not cause a lesion in only *n* = 1 mouse, thus not resulting in a significant change in neuroblasts (^*∗*^ *p* < 0.05).

Mouse brains stimulated at the low current density of 99 kC/m^2^ were stained for DCX to assess and quantify the effects of tDCS on neuroblasts in the SVZ (Figures 4(a)–4(c)). The area covered by DCX-positive neuroblasts was significantly enlarged in the ipsilateral SVZ following tDCS of either polarity (*p* < 0.05 [HSD]; Figure 4(d)). After cathodal tDCS, neuroblasts were even expanded in the SVZ contralateral to tDCS, suggesting a polarity-dependent degree of neuroblast-induction by tDCS (*p* < 0.05 [HSD]; Figure 4(d)). Additionally, neuroblasts in the SVZ were quantified in mice stimulated with 198 kC/m^2^ that did not display any lesion (*n* = 3 from the cathodal and *n* = 1 from the anodal group). After cathodal tDCS, neuroblasts were increased in both the ipsilateral and the contralateral SVZ. In the *n* = 1 anodally stimulated mouse without a cortical lesion, DCX-positive neuroblasts showed no significant difference to controls (Figure 4(e)).

### 3.4. Proliferation of Stem Cells in the SVZ Is Not Affected by tDCS

**Figure 5:**
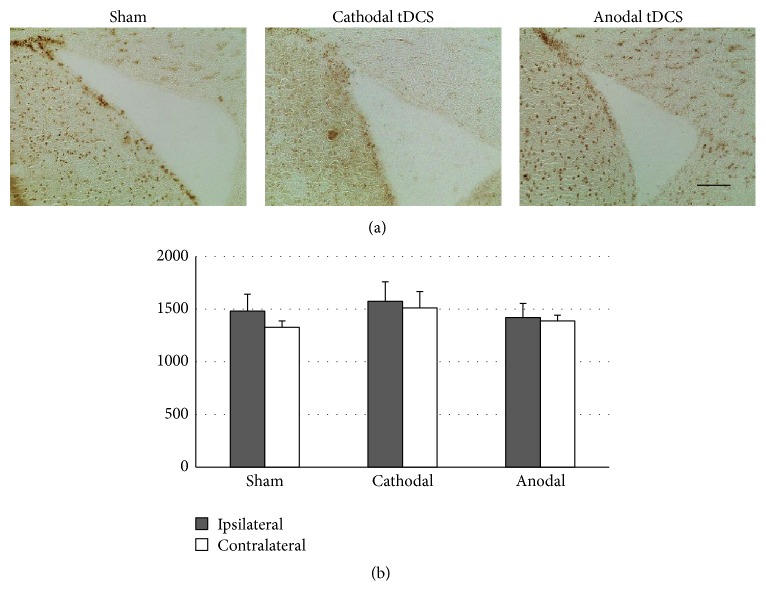
Multisession tDCS does not affect proliferation in the SVZ. (a) Proliferating cells in the SVZ were labeled with BrdU during anodal, cathodal, or sham tDCS. Immunohistochemistry revealed the size of the SVZ labeling positive for BrdU; the scale bar represents 100 *µ*m. (b) Multisession tDCS with 99 kC/m^2^ did not affect the size of the BrdU-positive area in the SVZ, suggesting that proliferation of stem cells was not changed by tDCS.

To assess the effect of tDCS on the proliferation of undifferentiated stem cells in the SVZ, animals were repetitively injected with BrdU during multisession tDCS. Staining for BrdU incorporation revealed that tDCS with 99 kC/m^2^ did not affect the amount of the BrdU+ stem cells in the SVZ (Figures 5(a)-5(b)).

## 4. Discussion

In a previous study, we methodologically established tDCS in the mouse and found that a single stimulation with a charge density of 198 kC/m^2^ or below did not cause lesions to the cortex. We here performed multisession tDCS in healthy mice for a total of 10 sessions, simulating a clinical rehabilitation paradigm. In this multisession setting, 50% of the mice did develop lesions to the cerebral cortex at a charge density of 198 kC/m^2^, but not at the lower charge density of 99 kC/m^2^. This indicates that the lesion threshold is lower for multisession tDCS than for single tDCS, suggesting a cumulative effect of the stimulation. Since not all mice develop lesions even at 198 kC/m^2^, we suggest stochastic effects within this dose range. Other effects of tDCS, such as sequence learning or pain perception, are known to be affected by the number of sessions as well, corroborating this cumulative response to stimulation [15, 16].

Until now, research on tDCS in experimental animals has mostly been limited to the rat [12, 13, 17–21], while only very few studies were performed in mice [22, 23]. Thus, tDCS effects need to be established in the mouse before genetically modified mice can be studied as a next step. In the rat, multisession tDCS of 128 kC/m^2^ does not cause any cortical lesions [12, 13]. Similarly, in the mouse, we here determined 99 kC/m^2^ as sublesional charge density and 198 kC/m^2^ to cause lesions in 50% of the mice. The different charge densities used in mice and rats result from the different sizes of the skull electrodes (2.27 mm^2^ for mice and 3.5 mm^2^ for rats). In humans, considerably lower charge densities of ~480 C/m^2^ are used typically. However, the strength of tDCS effects depends on the charge density [2, 24]. Thus, in this proof-of-principle study, the higher charge density was chosen to amplify the hypothesized effects of tDCS allowing for their characterization and to avoid missing any subtle effects.

Microglia activation goes along with increased expression of Iba1 antigen as detected by immunocytochemistry [25]. TDCS is capable of modifying microglia activation in cerebral cortex of mice and rats [13, 23]. In the rat, the number of Iba1-positive microglia is increased after both cathodal and anodal tDCS [13]. On the contrary, Peruzzotti-Jametti et al. suggested cathodal tDCS to reduce Iba1-positive microglia in the peri-ischemic cortex of mice when applied during acute focal cerebral ischemia, a condition that results in a robust activation of microglia [23]. Beyond this, we report on a focal decrease of Iba1 below the constitutive expression level. Thus, in the mouse, tDCS induced a polarity-dependent downregulation of Iba1, while in the rat, tDCS upregulates Iba1-expression. The current scarcity of experimental studies on microglia modulation by tDCS yet prohibits any comprehensive conclusion. Recent studies reveal differently polarized phenotypes of microglia, M1 and M2, suggesting heterogeneous microglia subpopulations with diverging surface marker profiles [26, 27]. Since Iba1 is expressed only in activated, not in resting, microglia [28], both up- and downregulation of Iba1 give evidence for a functional modification of microglia subpopulations by tDCS. Data suggest that animal species, stimulation polarity, and the existing level of inflammation due to a cortical lesion all influence the specific effects of tDCS on microglia. Further studies are needed to assess the effects of tDCS on microglia in regard of genomics, proteomics, and their interactome.

Brain-resident neural stem cells in the neurogenic niche of the SVZ were affected by both cathodal and anodal tDCS, leading to an increase in young neuroblasts. Similarly, tDCS mobilizes neural stem cells in the rat, leading to proliferation [13] and enhanced mobility [12]. After focal cerebral ischemia, neural stem cells migrate towards the lesion and promote regeneration by exerting pleiotropic effects [8, 9, 29]. Thus, it is conceivable that tDCS facilitates stroke rehabilitation via the modulation of microglia activation and neurogenesis.

## 5. Conclusions

TDCS elicits its actions through multifacetted mechanisms, far exceeding its primary effects on resting membrane potential. We here show that anodal tDCS downregulates constitutive expression of Iba1 on microglia in the cortex of the mouse, suggesting immunomodulatory effects. Moreover, cathodal, more than anodal, tDCS induced neurogenesis, supporting the use of tDCS in facilitating regeneration and recovery of function after stroke.

## Figures and Tables

**Table 1:** Overview over the experimental groups.

Current (C) charge density (*σ*)	Multisession cathodal tDCS	Multisession anodal tDCS	Control (no tDCS/sham)
C: 250 *µ*A *σ*: 99 kC/m^2^	*n* = 4	*n* = 4	*n* = 4
C: 500 *µ*A *σ*: 198 kC/m^2^	*n* = 4	*n* = 4	
